# Temporal network of experience sampling methodology identifies sleep disturbance as a central symptom in generalized anxiety disorder

**DOI:** 10.1186/s12888-024-05698-z

**Published:** 2024-03-29

**Authors:** Jiaxi Peng, Shuai Yuan, Zihan Wei, Chang Liu, Kuiliang Li, Xinyi Wei, Shangqing Yuan, Zhihua Guo, Lin Wu, Tingwei Feng, Yu Zhou, Jiayi Li, Qun Yang, Xufeng Liu, Shengjun Wu, Lei Ren

**Affiliations:** 1 Mental Health Education Center, Chengdu University, 610106 Chengdu, China; 2 University of Amsterdam, 1018WB Amsterdam, the Netherlands; 3 Xijing Hospital, Air Force Medical University, 710032 Xi’an, China; 4 Brain Park, School of Psychological Sciences, Turner Institute for Brain and Mental Health, Monash University, 3800 Clayton, VIC Australia; 5 Department of Psychology, Army Medical University, 400038 Chongqing, China; 6 Department of Psychology, Renmin University of China, 100000 Beijing, China; 7 School of Psychology, Capital Normal University, 100089 Beijing, China; 8 Department of Military Medical Psychology, Air Force Medical University, 710032 Xi’an, China; 9 Military Psychology Section, Logistics University of PAP, 300309 Tianjin, China; 10 Military Mental Health Services & Research Center, 300309 Tianjin, China

**Keywords:** Generalized anxiety disorder, Network analysis, Experience sampling methodology, Multilevel vector autoregression, Sleep disturbance

## Abstract

**Background:**

A temporal network of generalized anxiety disorder (GAD) symptoms could provide valuable understanding of the occurrence and maintenance of GAD. We aim to obtain an exploratory conceptualization of temporal GAD network and identify the central symptom.

**Methods:**

A sample of participants (*n* = 115) with elevated GAD-7 scores (Generalized Anxiety Disorder 7-Item Questionnaire [GAD-7] ≥ 10) participated in an online daily diary study in which they reported their GAD symptoms based on DSM-5 diagnostic criteria (eight symptoms in total) for 50 consecutive days. We used a multilevel VAR model to obtain the temporal network.

**Results:**

In temporal network, a lot of lagged relationships exist among GAD symptoms and these lagged relationships are all positive. All symptoms have autocorrelations and there are also some interesting feedback loops in temporal network. Sleep disturbance has the highest Out-strength centrality.

**Conclusions:**

This study indicates how GAD symptoms interact with each other and strengthen themselves over time, and particularly highlights the relationships between sleep disturbance and other GAD symptoms. Sleep disturbance may play an important role in the dynamic development and maintenance process of GAD. The present study may develop the knowledge of the theoretical model, diagnosis, prevention and intervention of GAD from a temporal symptoms network perspective.

**Supplementary Information:**

The online version contains supplementary material available at 10.1186/s12888-024-05698-z.

## Background

Generalized anxiety disorder (GAD) is a chronic anxiety disorder characterized by excessive and uncontrollable worry. It is quite typical for GAD to be accompanied by other non-specific psychological and physical symptoms [1]. According to a global epidemiological study, the combined lifetime prevalence of GAD is 3.7% [2]. GAD patients often suffer from severe functional impairments and have high rates of psychiatric comorbidities (e.g., major depressive disorder) [2]. Although pharmacotherapies and psychotherapies can effectively alleviate the symptoms of GAD in about 50% of the patients, it is still unclear how to treat those patients who partly or even not at all respond to the treatments [3].

Such disappointments– not uncommon in clinical practices– prompt researchers to move from a static view, where the links between symptoms are at best correlational, to a dynamic view, where the many symptoms related to a certain mental disorder are assumed to interact and co-evolve over time [4]. This new way of examining the causal relationships between a suite of related psychological symptoms is the core premise of the network approaches to psychopathology [5]. In essence, network models of symptoms focus on the causal relationships between symptoms and encourage the consideration of how the vicious cycle among symptoms affect the development and maintenance of certain mental disorders [4, 6, 7]. This perspective aligns with contemporary theories of GAD, which emphasize the self-perpetuating nature of its symptoms (through causal relations among symptoms) and how the disorder is reinforced and maintained through feedback loops [8]. For instance, according to the Metacognitive model (MCM), GAD may be maintained and perpetuated through a series of causal interactions between two types of worry. An individual might initially experience excessive worry about a potential negative outcome (Type 1 worry). This worry could lead to restlessness and muscle tension as the bodily reactions to perceived threats. As this worry persists, the individual begins to worry about the fact that they are always worrying (Type 2 worry) [9]. They might believe that this uncontrollable worry is harmful and indicative of a lack of mental control, leading to difficulty concentrating and irritability due to the constant self-monitoring and self-criticism. This, in turn, may cause sleep disturbances (worrying at night), leading to fatigue the following day. The fatigue and lack of sleep might then exacerbate the original worry, creating a feedback loop where each symptom feeds into and exacerbates the others. Similarly, according to the Emotion Dysregulation Model, heightened emotional arousal (which is frequently experienced among individuals with GAD) [10] may lead to irritability, which then triggers excessive worry (as maladaptive attempts to regulate the emotions) and restlessness [11]. As worry is oftentimes ineffective in managing the negative emotions, individuals may become anxious about their inability to manage the emotions and fuel the negative emotional arousal.

Network analysis offers a powerful tool for visualising and analysing the symptom-symptom interactions and feedback loops proposed in the contemporary theories of GAD. In a network model, each symptom of GAD is represented as a node in a network, and the causal influences between these symptoms are depicted as edges connecting the nodes. This structure allows researchers to directly map and analyse complex feedback loops where symptoms can reciprocally affect and perpetuate each other over time [12]. For instance, researchers may pinpoint how excessive worry leads to sleep disturbances, which in turn exacerbate fatigue and irritability. These symptoms then feed back into increased worry. This may address the inherent limitations of other modelling techniques based on latent variable frameworks (dynamic structural equation modelling), which often assume a common cause gives rise to symptoms, and by definition, do not contain feedback relations [12]. By neglecting the possibility of such feedback loops and reciprocal interactions among symptoms, these models potentially oversimplify the mechanistic processes in GAD. Furthermore, network analysis may unfold the most impactful and predominant symptoms underlying a certain mental disorder, and therefore offer clinical guidance for the design of effective treatments: the direct treatment on the most dominant symptom(s) may efficiently decrease or even stop the co-developments of other related symptoms. This view has also received support from empirical analysis: for example, Elliott and colleagues found that the symptoms with the most impact on the symptom network of anorexia nervosa at baseline were also mostly indicative of recovery [13]. Following this line of reasoning, the network approach is especially attractive for researchers to identify the most significant symptoms for GAD and thereby develop effective interventions.

At present, three studies established the network consisting of only anxiety symptoms [14–17] and the most of the anxiety symptom networks are based on anxiety-depression comorbidity [18–29], or other variables together with anxiety and depression, such as post-traumatic stress disorder [30, 31], eating disorder [32, 33], somatic symptomatology [34], intolerance of uncertainty [35], attention control [36], emotion regulation [37, 38], COVID-19-related variables [39, 40]. Most of the aforementioned studies used cross-sectional dataset and considered the cross-sectional design as one of their major limitations, as it failed to manifest temporal dynamic development and maintenance process of GAD symptoms and its results had to be interpreted with due caution. In addition, these networks were based on different questionnaires, such as State-Trait Anxiety Inventory-Trait subscale [16, 21, 33], Generalized Anxiety Disorder 7-Item Questionnaire (GAD-7) [14, 15, 17, 20, 23, 25–30, 35, 38], and Beck Anxiety Inventory [32, 37]. The symptoms based on these questionnaires only partially matches the symptoms diagnostic criteria of GAD based on the Diagnostic and Statistical Manual of Mental Disorders, the fifth Edition (DSM-5) [1], which is considered as the most definitive manual for diagnosis and treatment. For example, two GAD-7 symptoms (nervousness or anxiety and trouble relaxing) do not map to DSM-5 and four DSM-5 symptoms (being easily fatigued, difficulty concentrating or mind going blank, muscle tension and sleep disturbance) do not exist in GAD-7 [1, 41]. The disparities between DSM-5 and the other questionnaires limit the contribution of the aforementioned studies to the diagnosis, prevention and intervention of GAD. To address these limitations, the current study aims to directly assess the symptom network of GAD based on DSM-5 criteria.

The aim of this study is to establish an exploratory empirical conceptualization of temporal networks of GAD symptoms in order to clarify how the symptoms of GAD interact with each other and strengthen themselves over time. To estimate such temporal networks, we employ Experience sampling methodology (ESM) to conduct daily data collection over a period of 50 consecutive days. ESM has been used in investigating temporal dynamics among symptoms of different psychiatric disorders, including PTSD [42], depression [43], and eating disorders [44]. In ESM, some devices, such as handheld computers and smartphones, are often used for repeatedly collecting data from participants’ daily lives [45, 46]. This method has many advantages, including higher ecological validity and accuracy, smaller recall bias, and capability to identify changes of variables over time and dynamic relationships among variables [45, 47].

The recent innovation of statistical models has enabled the use of network models in the analysis of intensive longitudinal data gathered through ESM [48, 49]. Some of these models can be used to analyze data from a single individual (e.g., vector autoregression models; VAR) [50, 51], while others are designed for data collected from multiple individuals (e.g., multilevel VAR) [48, 52–54]. Of particular importance is the temporal network, which captures lagged relationships between symptoms from one time point (t-1) to the next (t), using Granger causality [55]. This type of network reveals how symptoms interact with each other and strengthen over time. Moreover, by examining the strength centrality (i.e., Out-strength and In-strength) of symptoms in the temporal network, researches can gain insights into the roles these symptoms play in the dynamic evolution of the symptom network system. The symptom with the highest Out-strength has the best ability to predict other symptoms in the next time point, while the symptom with the highest In-strength is predicted, to the greatest extent, by other symptoms in the previous time point.

In the present study, we estimated a multilevel network model based on daily diary data from individuals with elevated GAD-7 scores (i.e., GAD-7 ≥ 10) in order to investigate how symptoms of GAD, as defined by DSM-5 criteria, interact with each other and strengthen over time. This approach aims to enhance our understanding of the theoretical model, diagnosis, prevention and intervention of GAD from a temporal symptoms network perspective.

## Methods

### Participants and ethical statement

1062 (55% male) undergraduate students from the Fourth Military Medical University voluntarily completed the initial measure of GAD-7, a widely used tool for screening GAD and evaluating its severity [41]. From these students, 115 potential participants with a sum-score greater than a clinical cut point (GAD-7 ≥ 10 according to Spitzer et al. 2006 [41] and without medical history of mental disorder were preliminarily selected to take part in our study. We subsequently contacted this target group and informed them of the design and purpose of the subsequent daily diary study. All participants– among them 52% were male - agreed to participate in the study and gave consent for their participations. With an average age of 19.60 (SD = 1.02) and an average year of education amounting to 14.10 (SD = 0.78), participants recorded an average score of 12.16 (SD = 2.46) on the GAD-7.

The independent Ethics Committee, First Affiliated Hospital of Fourth Military Medical University granted ethical approval for the present study (Number: KY20182047-F-1). The daily questionnaire, administrated and collected via Wenjuanxing (www.wjx.cn), consisted of 20 items, in which only the first eight items were relevant to our study purpose and hence included in the current analysis. After finishing the questionnaire for each day, participants were given 3 RMB (about 0.4 US dollars) for compensation. Participants who had completed the questionnaire for more than 40 days were rewarded by double compensation. With an average completion of 47.66 days (SD = 4.28 days; range = 27–50) and the fact that no one has filled out the questionnaire for fewer than 25 days, participants were considered mostly cooperative in the current study.

### Procedure and measures

The time period of data collection was from June 8th, 2019 to July 26th, 2019. During the investigation, questionnaires were sent to participants at 20:00, with a deadline for responses set at 3 a.m. the next day [43]. Any data received after the deadline were treated as missing.

In this study, the eight daily self-reported symptoms under investigation are based on the diagnosis criteria of GAD in DSM-5 [1]. The set of symptoms includes two core symptoms: “Today, to what extent did you experience excessive anxiety and worry about a number of events or activities?” (excessive worry), and “Today, to what extent did you feel difficult to control the worry?” (uncontrollable worry). Besides, the set also includes six other symptoms: “Today, to what extent did you experience restlessness or a feeling of being keyed up or on edge?” (restlessness); “Today, to what extent did you feel easily fatigued?” (fatigue); “Today, to what extent were you having difficulty concentrating or mind going blank?” (difficulty concentrating); “Today, to what extent did you experience irritability?” (irritability); “Today, to what extent did you experience muscle tension?” (muscle tension); “To what extent did you experience sleep disturbance (difficulty falling or staying asleep, or restless, unsatisfying sleep) last night?” (sleep disturbance). Participants used a Likert scale to evaluate the severity of each GAD symptom, ranging from 1 (not at all) to 7 (very much) [43]. The questionnaire composed of these eight items was administrated in Chinese, with a formal back-translation procedure performed by two independent language experts. To prevent the potential confounding effects of careless responses, an attention check question was inserted in the questionnaire (i.e., “Today, please choose 1 for this item.”), and responses with an incorrect answer to this question were deemed invalid [56]. The average daily internal consistency reliability for the GAD symptoms composite was 0.87 (Cronbach’s alpha), ranging from 0.80 to 0.93.

### Data analysis

With responses collected for 50 consecutive days, the current study can be considered well-powered, based on the results of a series of simulations [50]. As discussed in the introduction, the multilevel vector autoregressive model, implemented in the package *“mlVAR”* [48], was employed to reveal the complex and dynamic system of the eight GAD systems. However, a notable limitation of mlVAR is its inability to handle missing data. To ensure the robustness of the current findings, two pre-processing strategies were employed to address missing responses prior to the mlVAR analysis. First, given the small amount of missing data (for the 8 ESM variables over 50 days, 1140 of the 46,000 data points (2.48%) were missing) and the lack of indication that these missing data did not emerge randomly, we did not apply any data imputation techniques but instead utilized the list-wise deletion strategy [43]. The results obtained from this strategy were reported below. The second strategy we employed is to impute missing entries in our time series data using the moving average method, which is considered one of the best methods for imputing missing data in time series. The results obtained from this strategy were detailed in the supplementary material. We then carried out the Kwiatkowski-Phillips-Schmidt-Shin test on each of our study variable and found that every variable fulfilled the requirement of stationary assumption. Moreover, these data also fulfilled the assumption which required the same time lag in a consecutive assessment.

The multilevel VAR allows slopes and intercepts to change between participants for the purpose of explaining possible interindividual differences. In a temporal network, a directed edge with an arrow connecting two nodes indicates the lagged relationship between these two nodes from one time point to the next. The direction of arrows represents the direction of the lagged relationship. In this study, we constrained random effects of incoming edges to a single node (in the temporal networks) to be orthogonal, such that the models are estimable. In temporal networks, an “and” rule is used to select significant edges: an edge is retained if both regressions on which the edge is based are significant (α = 0.05). Moreover, we also estimated contemporaneous network with undirected edges to captured the contemporaneous associations between these GAD symptoms. Contemporaneous associations have been understood as dynamics that may occur more limited timescale (e.g., within a few hours) than those obtained in the temporal network (i.e., daily in the current research) [50]. In the present study, these contemporaneous effects depict relationships emerging on the same day while temporal network describes the dynamics of variables on a day-to-day basis.

For the temporal network, it is needed to calculate the Out-strength and In-strength of each node. The Out-strength of a node refers to the absolute sum of weights of edges pointing to other nodes in the network, indicating the ability of a symptom affecting other symptoms in the next time point. The In-strength of a node refers to the absolute sum of weights of edges pointing to this node in the network, representing the extent of a symptom affected by other symptoms in the previous time point [49]. For the contemporaneous network, strength centrality was estimated by adding up the absolute edge weights of all edges linked with a node. This calculation reflects the overall extent of the connectivity of a specific node within the network [57, 58]. In accordance with the reporting standards in the field of network analysis, all centrality indices were presented using raw scores [59].

The results of inter-individual differences in temporal network [60–62] and codes used in the analysis process can be found in the Supplementary Materials.

## Results

The range, average scores and standard deviations of individual symptoms are depicted in Table 1. Among all of these symptoms, fatigue and excessive worry have the highest severity while muscle tension and irritability have the lowest.

**Table 1:** Descriptive statistics for individual symptoms in overall sample (*N* = 115)

Symptom	Range	Mean	SD	Out-strength	In-strength
Excessive worry	1–7	3.91	1.60	0.10	0.32
Uncontrollable worry	1–7	3.67	1.69	0.17	0.33
Restlessness	1–7	3.43	1.67	0.08	0.32
Fatigue	1–7	4.02	1.70	0.23	0.50
Difficulty concentrating	1–7	3.65	1.69	0.14	0.32
Irritability	1–7	3.03	1.67	0	0.19
Muscle tension	1–7	2.82	1.55	0.04	0.19
Sleep disturbance	1–7	3.73	1.83	1.41	0

*Note* Out-strength represents the sum of absolute values of significant edges from a given symptom (excluding autocorrelation) and In-strength represents the sum of absolute values of significant edges to a given symptom (excluding autocorrelation) in the temporal network

**Fig. 1:**
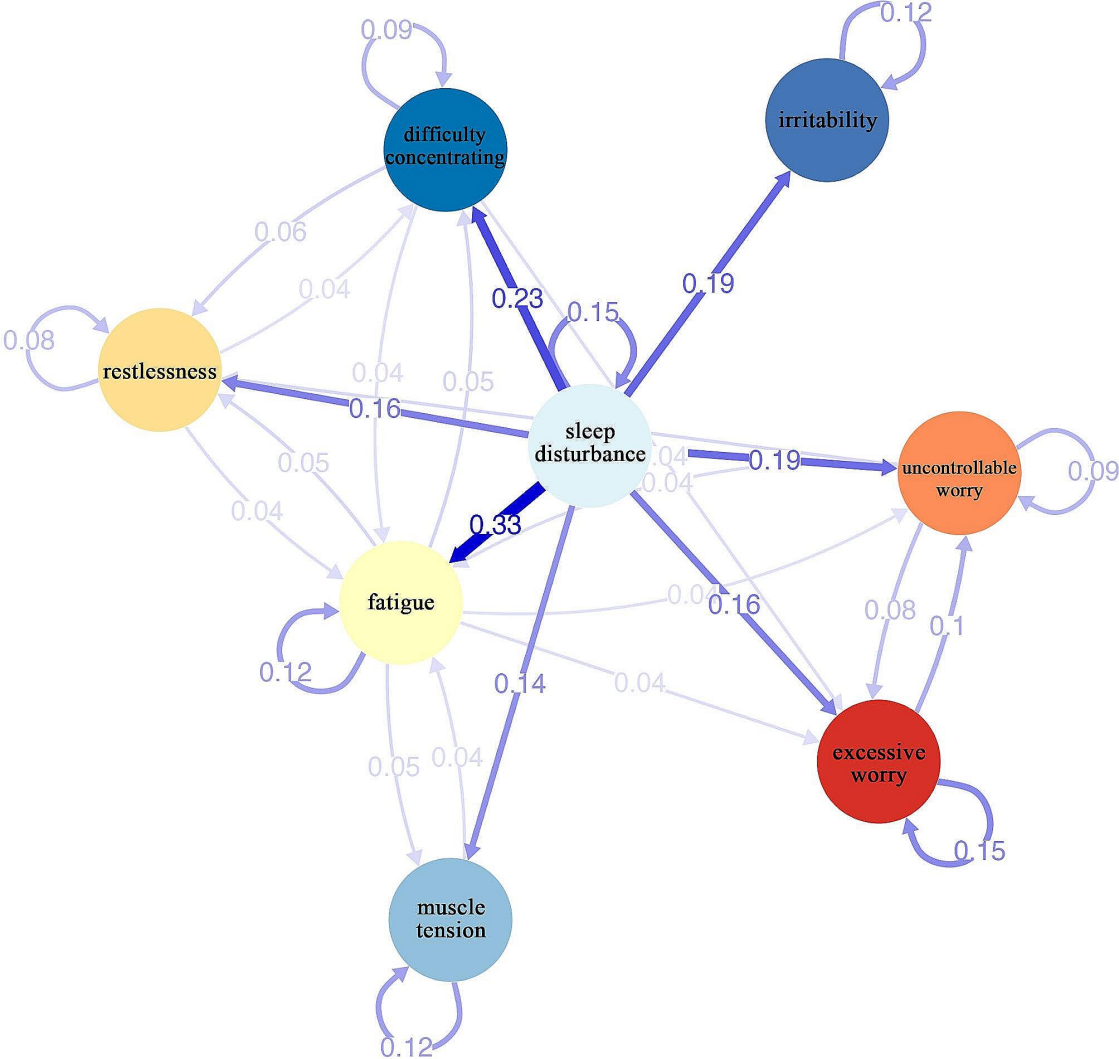
Temporal networks of generalized anxiety disorder symptoms. *Note * Blue edges represent positive relationships between nodes, red edges represent negative relationships between nodes. Thicker edges between nodes represent stronger relationships. The numbers represent significant edge weights

The temporal network is shown in Fig. 1. There are several obvious features emerge when summarizing the temporal network. Firstly, 22 edges (excluding 8 autocorrelation edges) are not zero (39%) among 56 possible edges (excluding autocorrelation edges) and all of these edges are positive. Secondly, sleep disturbance has Granger causal (predictive) effects on all other seven symptoms and the lagged relationships from sleep disturbance to fatigue (weight = 0.33), difficulty concentrating (weight = 0.23), irritability (weight = 0.19) and uncontrollable worry (weight = 0.19) have the highest weights. Thirdly, all of these eight symptoms have autocorrelations (edges pointing toward themselves). This means that all these symptoms have Granger predictive effects on themselves. Sleep disturbance (weight = 0.15) and excessive worry (weight = 0.15) have the highest autocorrelations. Finally, there are some interesting feedback loops in the temporal network. For example, excessive worry has predictive effect on uncontrollable worry (weight = 0.10) which in turn has predictive effect on excessive worry (weight = 0.08). Fatigue predicted muscle tension (weight = 0.05) which in turn predicted fatigue (weight = 0.04). There are bidirectional feedback loops among restlessness, fatigue and difficulty concentrating, and also feedback loops between either two of them (specific weights see Fig. 1). The contemporaneous network is shown in Fig. S3. Strong associations are found between uncontrollable worry and excessive worry (weight = 0.48), uncontrollable worry and restlessness (weight = 0.25), excessive worry and restlessness (weight = 0.25), fatigue and difficulty concentrating (weight = 0.24), and irritability and muscle tension (weight = 0.23).

**Fig. 2:**
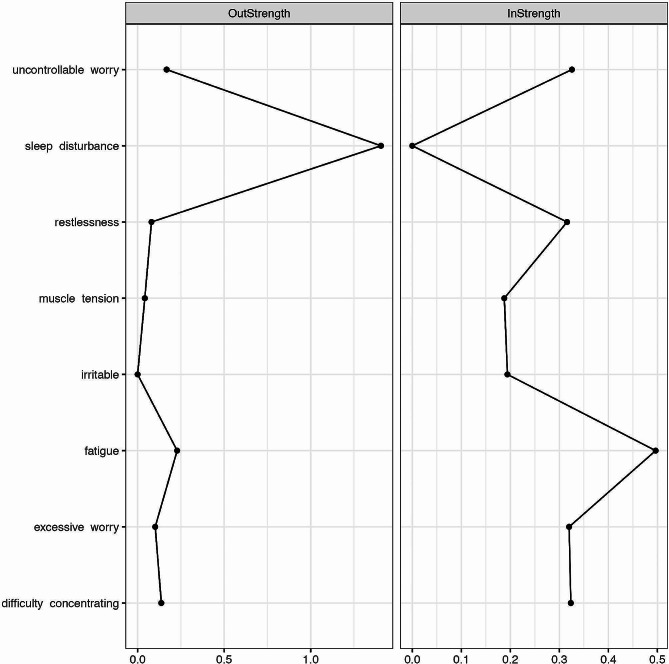
Strength centrality of generalized anxiety disorder symptoms within the temporal networks

The Out-strength and In-strength (excluding autocorrelation) of GAD symptoms in the temporal network are shown in Table 1; Fig. 2. Sleep disturbance has the obviously highest Out-strength among these symptoms, indicating the predictive effect of this symptom on other symptoms in the next time point is strongest. In other words, the more sleep disturbance a participant has at one time point, the more likely the participant is to report other symptoms of GAD at the next time point. Irritability has the lowest Out-strength (value = 0) among these symptoms, indicating this symptom does not have predictive effect on any other symptoms. Meanwhile, fatigue has the highest In-strength among these symptoms, which means that this symptom could be predicted, to the largest effect, by other symptoms in the former time point. Sleep disturbance has the lowest In-strength (value = 0) among these symptoms, indicating this symptom could not be predicted by any other symptoms in the former time point. The strength centrality of GAD symptoms in the contemporaneous network is shown in Fig. S4. Uncontrollable worry has the highest overall connectivity in the network, followed by restlessness and excessive worry.

The temporal network estimating by dataset which used moving average imputation strategy is shown in Fig. S1 (in the Supplementary Materials). Fig. S3 (in the Supplementary Materials) shows the Out-strength and In-strength (excluding autocorrelation) of GAD symptoms. In fact, the results of temporal networks estimating by dataset which used moving average imputation strategy and list-wise deletion strategy (i.e., did not apply any data imputation techniques) are very similar. This further proves the robustness of the analysis.

## Discussion

To the best of our knowledge, this is the first article exploring the dynamics internal structure of GAD symptoms based on DSM-5 diagnostic criteria by using daily life data from participants with elevated GAD-7 scores. The temporal network could unfold how the symptoms of GAD interact with each other and strengthen themselves over time on an average scale. Meanwhile, the strength centrality could cast light on which symptoms may play an important role in the dynamic development and maintenance process of GAD.

In temporal network, a lot of lagged relationships exist among GAD symptoms and these lagged relationships are all positive. These results may support the network theory of mental disorders which pointed out that mental disorders arise from direct interactions between symptoms [4]. Sleep disturbance has predictive effects on all other GAD symptoms and the lagged relationships from sleep disturbance to fatigue and difficulty concentrating have the highest weights. The lagged relationships between sleep disturbance and these two symptoms are frequently observed in empirical settings and may be interpreted as: the more sleep disturbance the person has in the former night, the more fatigue and difficulty concentrating he might have in the next day [63]. Previous studies have shown that individuals with sleep disturbance, such as insomnia, exhibit evidence like increased hypothalamic-pituitary-adrenal activity [64], sympathetic tone [65] and daytime arousal [66, 67]. These changes would lead to a higher likelihood of activating cognitive, emotional, and behavioral symptoms of GAD, such as irritability, restlessness, muscle tension, instability of thinking activities and emotions, and so on [68, 69]. In fact, there are many studies using different research methods that indicate sleep disturbance can predict anxiety [70–73]. Based on our knowledge, the contemporary theories of GAD rarely mention the role of sleep disturbance in the development and maintenance of GAD [8]. An important contribution of the current research is the first investigation of the temporal network of GAD symptoms based on DSM-5, which identified a widespread predictive effects of sleep disturbance on other GAD symptoms. Such findings may facilitate further development of GAD theoretical models.

In accordance with previous temporal studies of psychopathological dynamics [42, 43, 57], all symptoms in temporal network had autocorrelations. This means that these symptoms can predict themselves from one time point to the next time point. This may be the first indication of a ‘critical slowing down’, because of the gradual crystallization of pathological responses [74].

Some vicious cycles also deserve special attention. Consistent with previous dynamic network analysis studies including excessive worry and uncontrollable worry [57, 58], the current study finds that excessive worry has predictive effects on uncontrollable worry and uncontrollable worry also has predictive effects on excessive worry. In other words, excessive worry and uncontrollable worry can strengthen each other over time, which may lead to more severe clinical symptoms. This is understandable as that when individuals feel difficult to control the worry, they will worry about a number of events or activities, vice versa. In line with prior articles [57, 58], a strong contemporaneous association between these two symptoms is also detected within the same day highlighting how excessive worry and uncontrollable worry may aggravate each other on a closer time scale. This finding supports the MCM of GAD (more details see Introduction section), which emphasizes the causal interactions between Type 1 worry (i.e., excessive worry) and Type 2 worry (e.g., uncontrollable worry) [9, 75, 76]. The same type of loops also appears between fatigue and muscle tension, and among restlessness, fatigue and difficulty concentrating. These loops may provide important ways for us to understand the development and maintenance of GAD. Further studies are needed to understand these loops.

Strength centrality results show that sleep disturbance has the obviously highest Out-strength and lowest In-strength among GAD symptoms. Thus, sleep disturbance of the previous day has a great ability to predict all the other symptoms on the next day. This result indicates that sleep disturbance may play an important role in the dynamic development and maintenance process of GAD. A recent study showed that having a full night of sleep is helpful in remitting anxiety and mood-stabilizing, while a lack of sleep causing anxiety levels to rise by as much as 30% [73]. Additionally, strength centrality results show that fatigue has the highest In-strength. Thus, fatigue is greatly affected by other symptoms in the previous time point. This might be one of the reasons for its high severity. Moreover, uncontrollable worry has the highest overall connectivity in the contemporaneous network, indicating its central role in GAD various symptoms on a within-day basis. Thus, this result provides some evidence that the MCM provides a good theoretical framework for conceptualizing GAD [9, 75].

Due to sleep disturbance predicts all other GAD symptoms at the next time point and it has the obviously highest Out-strength among GAD symptoms, it is vital to reconsider the importance of sleep disturbance: the present study probed that sleep disturbance may not be merely an accompanying symptom of GAD but an important cause of GAD symptoms from a temporal network perspective. A recent review also pointed out that sleep disturbance is likely to be a contributory causal factor in the occurrence of most mental health conditions [77]. Thus, it may be reasonable for a clinical worker to take sleep disturbance as an important target for intervening, in contrast to the current mainstream treatment of psychiatric disorders which considers the treatment of sleep disturbance as an afterthought in patient care [77]. In other words, having a good sleep may be an excellent solution for GAD. Recent treatment efforts have also shed light on the benefit of treating sleep disturbance before other symptoms among patients with various psychiatric disorders [77]. As a multi-component and evidence-based treatment [78], cognitive behavioral therapy for insomnia (CBT-I) has been highlighted as the first choice for patients with insomnia disorder in recent treatment guidelines [79–81]. It is encouraging to note that an open trial study suggests that CBT-I is an effective therapy for patients with co-morbid insomnia disorder and GAD [82]. More clinical treatment studies can be conducted in the future. In addition, these results may imply the importance of sleep disorders in the diagnosis of GAD. It is worth mentioning that GAD-7, as a widely used tool for screening GAD and evaluating its severity [41], does not include sleep disturbance item, which may reduce its screening and evaluation performance. This still needs further exploration. Finally, considering the highest overall connectivity of uncontrollable worry in the contemporaneous network, the uncontrollability of worries should also be considered a priority target, similar to metacognitive therapy for GAD [83–85].

Some limitations are noteworthy in the present study. First of all, multilevel VAR models are complex with a large number of parameters to be estimated, therefore a relatively large sample size (including both the number of observations and the number of measurements per observation) is required for stable and consistent estimation [86]. According to previous methodological review and simulation studies [86, 87], the number of observations (115) analyzed in the current study and the number of measurements per observation (50) are close to the medium level (i.e., 100 for the number of observations and 60 for the number of measurements) and can lead to robust estimations of the multilevel VAR model. We also encourage future research use different samples in other settings (e.g., observations coming from different countries or different cultures) to further examine the robustness of our findings. Second, instead of patients with a definite diagnosis of GAD, we recruited undergraduate students whose sum-score exceeded the clinical threshold of GAD screening tool (i.e., GAD-7) in this study. This may lead to a lack of representativeness of the clinical sample. Third, if the time window between assessments were different from the development of the actual relationship between symptoms, it might be hard to discover critical underlying relationships when purely based on the current set of analyses. For example, the predictive relationship between symptoms within a timescale of several hours might not be revealed from a network based on daily assessments. Fourth, there are many different manifestations of sleep disturbance (i.e., difficulty falling or staying asleep, or restless, unsatisfying sleep). So, it is not possible to find out how a specific manifestation of sleep disturbance affects other symptoms of GAD by using the item of general sleep disturbance. Restlessness (i.e., restlessness or a feeling of being keyed up or on edge) and difficulty concentrating (i.e., difficulty concentrating or mind going blank) also share this problem. For future studies, we encourage researchers to separate the many aspects of these symptoms and hence provide an even more detailed analysis of the interplay of symptoms. Last but not least, combining ESM with network models is a novel exploratory method, instead of a confirmatory method, which needs to be considered when interpreting current results [88].

## Conclusions

The present study is the first study combining experience sampling methodology with network analysis in exploring the temporal network of GAD symptoms based on DSM-5, using daily life data derived from participants with elevated GAD-7 scores. This study establishes a preliminary exploratory empirical conceptualization of how GAD symptoms interact with each other and strengthen themselves over time, and particularly highlights the relationships between sleep disturbance and other GAD symptoms. Sleep disturbance may play an important role in the dynamic development and maintenance process of GAD. The present study may develop the knowledge of the diagnosis, prevention and intervention of GAD from temporal symptoms network perspective.

### Electronic supplementary material

Below is the link to the electronic supplementary material.

Supplementary Material 1

## Data Availability

The datasets used during the current study are available from the corresponding author on reasonable request. The codes can be found in the Supplementary Materials.

## Abbreviations

GAD: Generalized anxiety disorder
MCM: Metacognitive model
GAD-7: Generalized Anxiety Disorder 7-Item Questionnaire
DSM-5: Diagnostic and Statistical Manual of Mental Disorders, the fifth Edition
ESM: Experience sampling methodology
VAR: Vector autoregression models
CBT-I: cognitive behavioral therapy for insomnia
