# Supplementary material for: Temporal network of experience sampling methodology identifies sleep disturbance as a central symptom in generalized anxiety disorder
Source: BMC Psychiatry. 2024 Mar 29;24:241. doi: 10.1186/s12888-024-05698-z (PMC10981297; doi:10.1186/s12888-024-05698-z)
Supplement: Supplementary file 1 — Supplementary Material 1 [file 12888_2024_5698_MOESM1_ESM.pdf]

Supplementary Materials

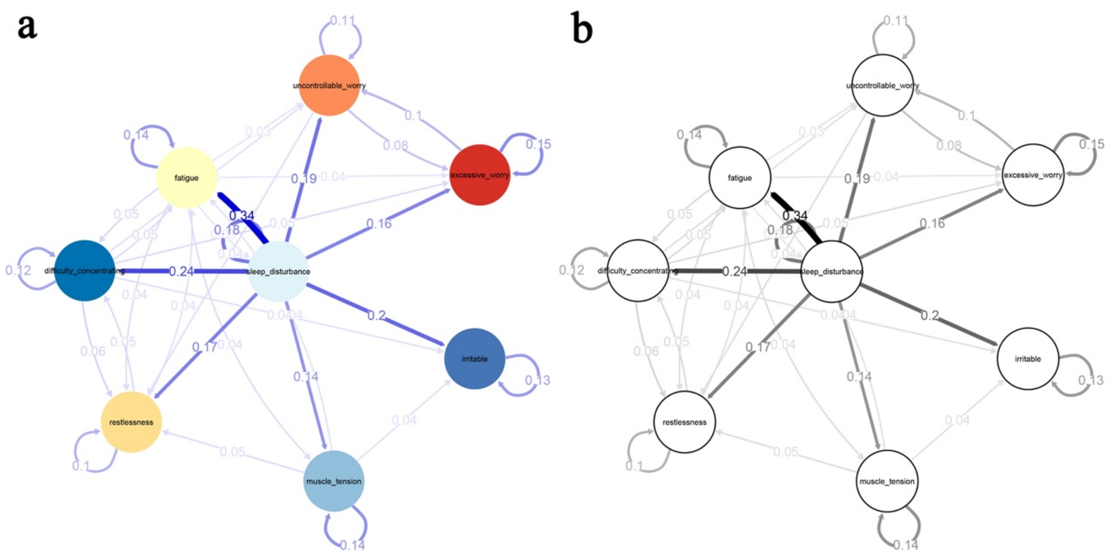

Fig. S1. Temporal networks of generalized anxiety disorder symptoms estimating by dataset which used moving average imputation strategy. (a) colorful mode; (b) black and white mode.

Note: Blue (a) and black continuous (b) edges represent positive relationships between nodes, red (a) and black discontinuous (b) edges represent negative relationships between nodes. Thicker edges between nodes represent stronger relationships. The numbers represent significant edge weights.

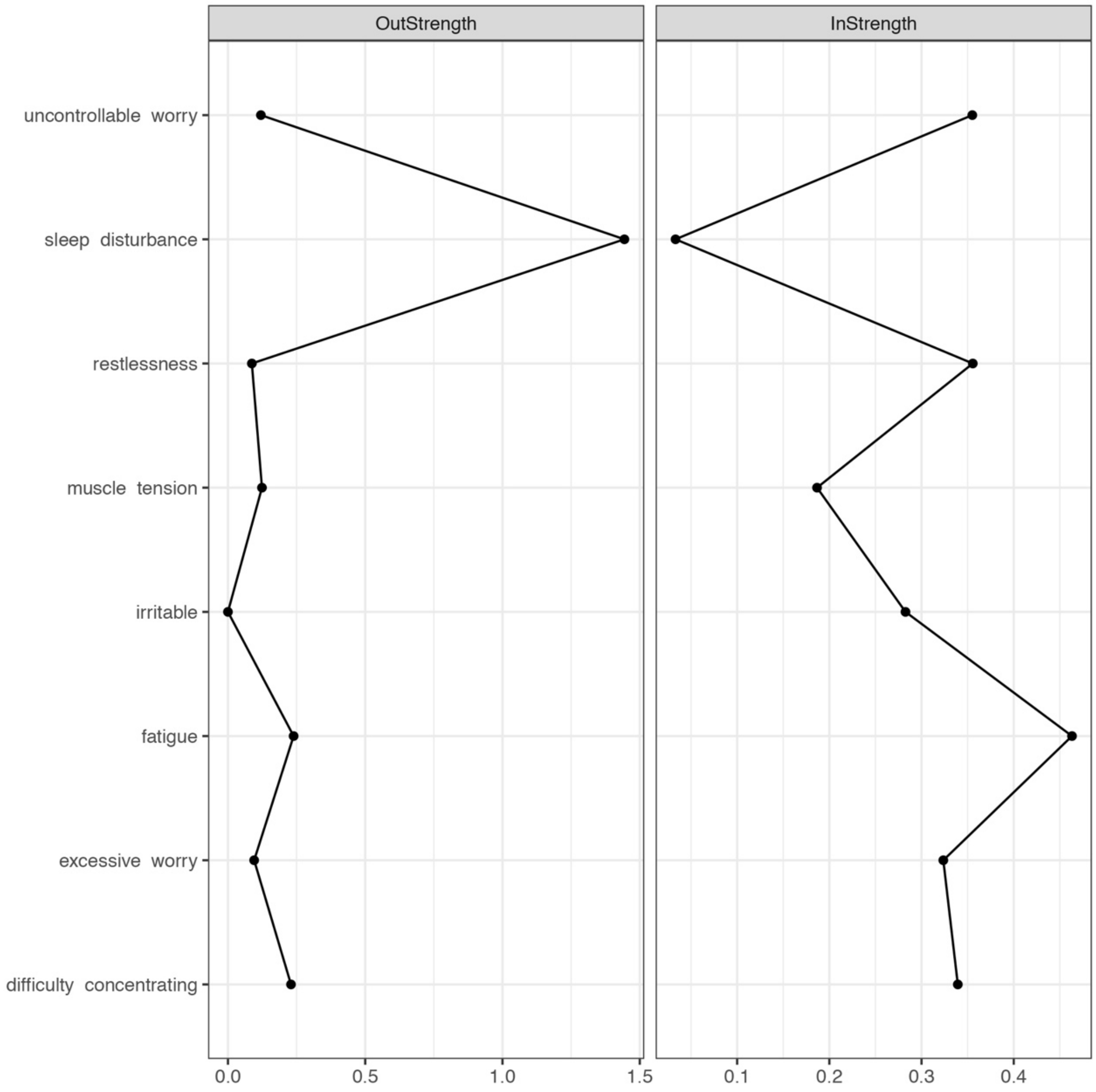

Fig. S2. Strength centrality of generalized anxiety disorder symptoms within the temporal networks estimating by dataset which used moving average imputation strategy.

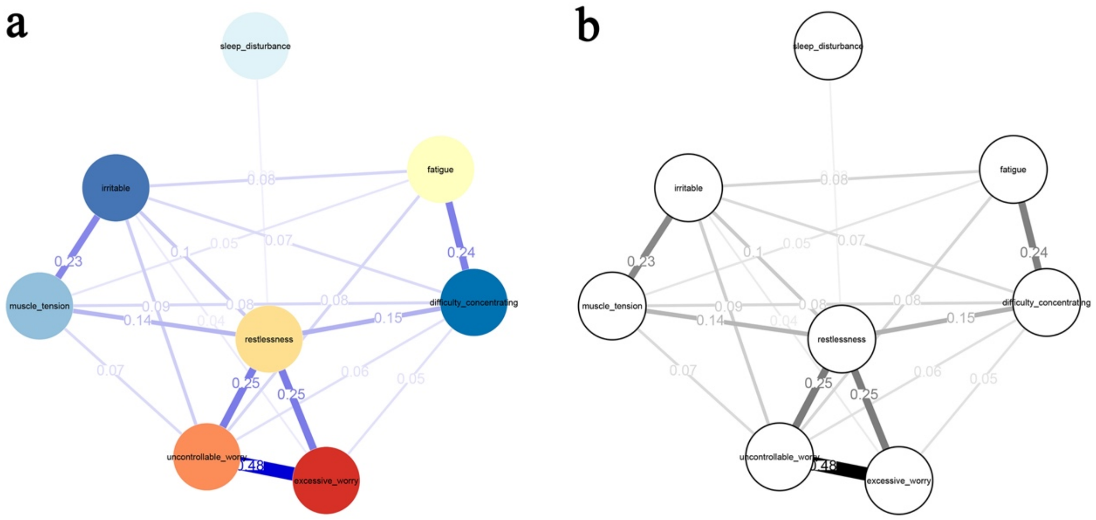

Fig. S3. Contemporaneous networks of generalized anxiety disorder symptoms. (a) colorful mode; (b) black and white mode.

Note: Blue (a) and black continuous (b) edges represent positive relationships between nodes, red (a) and black discontinuous (b) edges represent negative relationships between nodes. Thicker edges between nodes represent stronger relationships. The numbers represent significant edge weights.

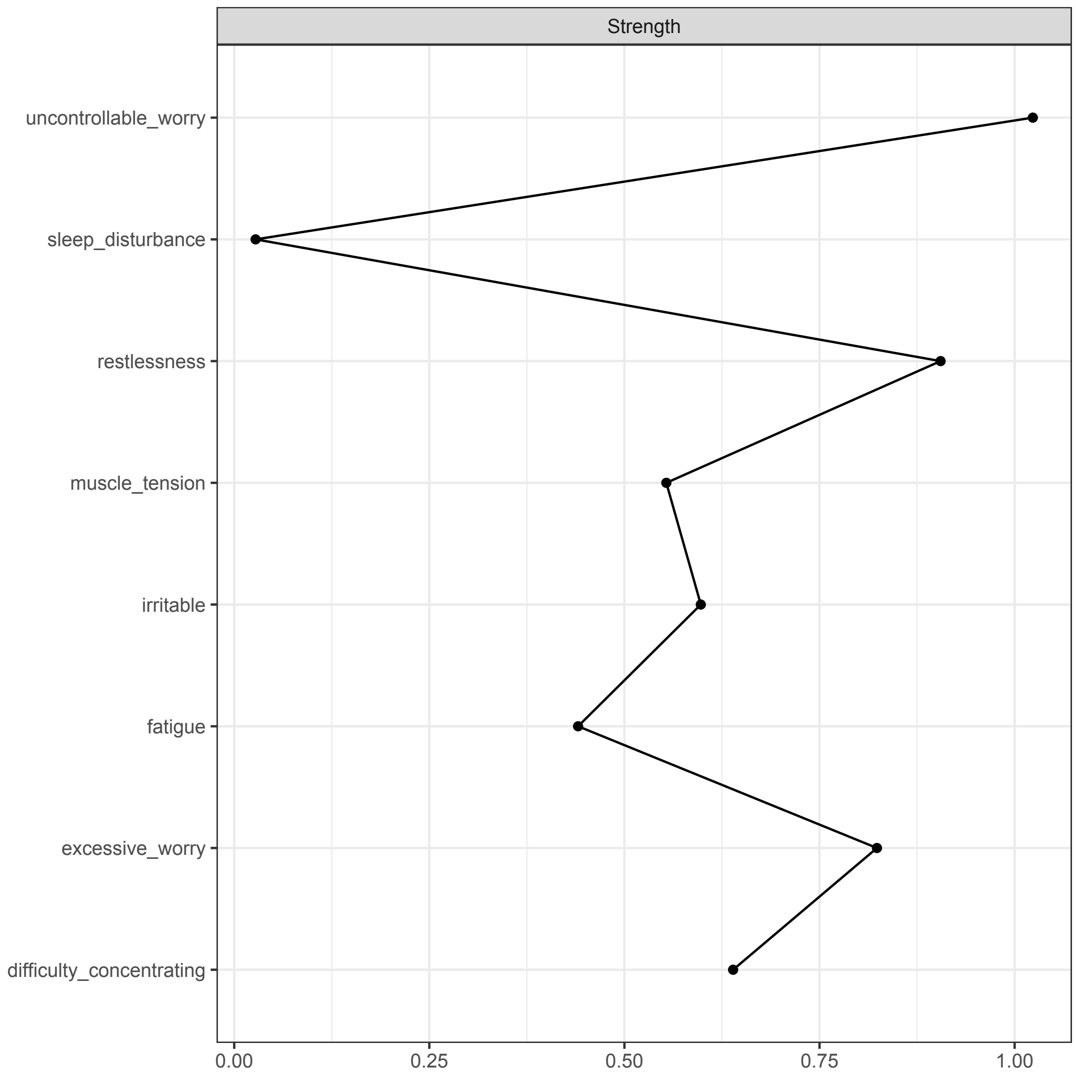

Fig. S4. Strength centrality of generalized anxiety disorder symptoms within the contemporaneous network.

Codes

```
library("tseries")

library(mlVAR)

library(qgraph)

library(apaTables)

library(dplyr)

library(reshape2)

library(imputeTS)

library(bootnet)

library(cluster)

library(mclust)

library(vars)


getwd()

setwd("/Users/renlei/Desktop/temporal network")

all.data.non1 <- read.csv(file = "maydata.csv")

vars.list1 <- c("excessive_worry", "uncontrollable_worry", "restlessness", "fatigue",

                        "difficulty_concentrating", "irritable", "muscle_tension",

"sleep_disturbance")

all.data.non1 <- as.matrix(all.data.non1)

colnames(all.data.non1)[2:9] <- vars.list1
```

```
non.results1 <- mlVAR(as.data.frame(all.data.non1[,c(2:9,11)]), vars = vars.list1,
idvar = "ind", lags = 1, estimator = "lmer", contemporaneous = "orthogonal",
temporal = "orthogonal")

graph1 <- plot(non.results1, type = "temporal", rule = "and", layout = "spring", vsize
= 10.5, label.cex = 0.6, label.scale = FALSE, edge.labels = TRUE, theme =
"colorblind", layoutScale = c(1,1), nonsig = "hide", asize = 3 ,mar =
c(4,4,4,4),border.width=0.3, border.color='000000', color=c("#d73027", "#fc8d59",
"#fee090", "#ffffbf","#0072B2","#4575b4","#91bfdb","#e0f3f8"))

graph2 <- plot(non.results1, type = "contemporaneous", layout = "spring",    vsize =
10.5, label.cex = 0.6, label.scale = FALSE, edge.labels = TRUE, theme =
"colorblind", layoutScale = c(1,1), nonsig = "hide", asize = 3 ,mar =
c(4,4,4,4),border.width=0.3, border.color='000000', color=c("#d73027", "#fc8d59",
"#fee090", "#ffffbf","#0072B2","#4575b4","#91bfdb","#e0f3f8"))

cont1 <- getNet(non.results1, "temporal", layout = "spring", nonsig = "hide", rule =
"and")
centralityPlot(graph1, scale = "raw",include = c("OutStrength","InStrength"))
centralityTable(graph1)
centralityPlot(graph2, scale = "raw",include = c("Strength"))
centralityTable(graph2)
```

```
####################
## the application of LCVAR as illustrated in Ernst et al., 2021
#################
all.data.non1 <- read.csv(file = "mydata.csv")
## the data set should be missing data imputed
vars.list1 <- c("excessive_worry", "uncontrollable_worry", "restlessness", "fatigue",
                "difficulty_concentrating", "irritable", "muscle_tension",
"sleep_disturbance")
all.data.non1 <- as.matrix(all.data.non1)
colnames(all.data.non1)[2:9] <- vars.list1
# Calculate VAR(p) model for each person
RegrModel_per_pers = matrix(0, 115, 72)
for(pers in 1:115){
  ind_obs = which(all.data.non1[,11] == pers)
  RegrCoeff_pers = matrix(0, 8, 9)
  v <- VAR(all.data.non1[ind_obs, 2:9], type = "const", #    const is to include an
intercept
           season = NULL, p = 1
  )
  Terms = length(v$varresult[[1]]$coefficients) - 1
```

```
  for(vas in 1:8){
    RegrCoeff_pers[vas, 1] = tail(v$varresult[[vas]]$coefficients, n = 1) #to have
const at beginning
    RegrCoeff_pers[vas, 2:(Terms+1)] = v$varresult[[vas]]$coefficients[1:Terms]
  }

  RegrModel_per_pers[pers, ] = as.vector(t(RegrCoeff_pers))
  # matrix(as.vector(t(RegrCoeff_pers)), ncol = nDepVar) # get a matrix of
regression coefficients
}

allfit <- matrix(0,5,50)
for(run in 1:50){
  mod <- Mclust(RegrModel_per_pers, G = 5,
                initialization = list(hcPairs = randomPairs(RegrModel_per_pers)))
  # mclust needs even number of objects to cluster
  # Make ProbRegrCoeff have same dimensions than RegrCoeff nVar+1 by
(nVAr*nCluster)
  # ProbRegrCoeff <- matrix(summary(mod, parameters = T)$mean , nrow =
(numberVaribales + 1))

  allfit[5,run] = summary(mod, parameters = T)$bic
```

```
}

## by checking the results of allfit, we can determine the best model

apply(allfit, 1, mean)
```

The results of inter-individual differences in temporal network

The series of studies by Ernst, Timmerman, Albers, and colleagues offered powerful tools to examine inter-individual (a.k.a. group and cluster) differences in vector autoregressive models [1–3]. Because its strong tendency to overfit and capture noise, cluster analysis is only appropriate with theoretically sound inferences on the numbers and patterns of cluster differences [4, 5], and as the first study examining the dynamic networks of the GAD symptoms, the current research unfortunately misses the substantiated theoretical assumptions on such cluster differences.

To entertain the possibility of a data-driven approach to determining the inter-individual differences in temporal networks, we report the results of latent class vector autoregressive model (LCVAR) [2], an advanced, probabilistic cluster approach for vector autoregressive analysis that strikes a balance between model complexity and flexibility. A dynamic clustering method, LCVAR essentially identify subgroups of people who are characterized by qualitatively similar symptoms dynamics. The analysis (the code of which is enclosed in the supplementary material as well) showed that, when using the value of BIC to determine the optimal number of clusters, 2-cluster solutions (BIC = 1542) are considered better than 3-cluster (BIC = 1849), 4-cluster (BIC = 2014), or 5-cluster (BIC = 1983) solutions. However, it is trickier to choose between the 2-cluster and 1-cluster solution by merely comparing the BIC values; once again, because of the lack of theory in this context that suggests

2-cluster solution, we determine to continue with the current report with the 1-cluster solution.

References:

1. Ernst AF, Albers CJ, Jeronimus BF, Timmerman ME. Inter-individual differences in multivariate time-series: latent class vector-autoregressive modeling. Eur J Psychol Assess. 2020;36(3):482–91.
2. Ernst AF, Timmerman ME, Jeronimus BF, Albers CJ. Insight into individual differences in emotion dynamics with clustering. Assessment. 2021;28(4):1186–206.
3. Ernst AF, Timmerman ME, Ji F, Jeronimus BF, Albers CJ. Mixture multilevel vector-autoregressive modeling. Psychol Methods. 2023; doi: 10.1037/met0000551.
4. Yuan S. Novel clustering methods for complex cluster structures in behavioral sciences (Doctoral dissertation, Tilburg University). 2022.
5. Vermunt JK, Magidson J. Latent class cluster analysis. Applied latent class analysis, 2002;11(89-106):60.
